# Supplementary material for: Comparative genomics reveals the dynamics of chromosome evolution in Lepidoptera
Source: Nat Ecol Evol. 2024 Feb 21;8(4):777–90. doi: 10.1038/s41559-024-02329-4 (PMC11009112; doi:10.1038/s41559-024-02329-4)
Supplement: Supplementary file 1 — Supplementary Text and Figs. 1–23. [file 41559_2024_2329_MOESM1_ESM.pdf]

nature ecology & evolution

Article

https://doi.org/10.1038/s41559-024-02329-4

Comparative genomics reveals the dynamics of chromosome evolution in Lepidoptera

In the format provided by the authors and unedited

Table of Contents

Table of Contents 0
List of Supplementary Tables 2
Supplementary Table 1: Genome assemblies information. 2
Supplementary Table 2: W chromosome information. 2
Supplementary Table 3: Gene annotation information. 2
Supplementary Table 4: Assignment of 4,112 single copy orthologues to Merian elements. 2
Supplementary Table 5: Inferred fusion and fission events with Syngraph. 2
Supplementary Table 6: Inferred fusion and fission events with LFFF. 3
Supplementary Table 7: Inferred fusion and fission events with Syngraph for highly rearranged species. 3
Supplementary Table 8: Genomic correlates of chromosome length across 193 species. 3
Supplementary Table 9: Mean feature density per Merian element across the dataset. 3
Supplementary Table 10: Feature statistics for all 6,254 chromosomes in the dataset. 4
Supplementary Table 11: Proportion of each genome annotation by the main TE classifications. 4
Description of files contained in the Zenodo repository 5
Supplementary Text 5
Section 1. Dataset selection 5
Section 1. Identification of transposable elements 5
Section 1. Phylogeny 6
Section 2. Assignment of orthologues to Merian elements 6
Section 3. Filtering the dataset when inferring fusion and fission events 6
Supplementary Figures 8
Supplementary Figure 1. Assessing quality of gene annotations using the number of missing conserved single-copy genes. 8
Supplementary Figure 2. Assessing quality of gene annotations using the number of duplicated conserved single-copy genes. 9
Supplementary Figure 3. Distribution of haploid chromosome number in Lepidoptera from 210 species. 10
Supplementary Figure 4. Distribution of genome size (Mb) in Lepidoptera from 210 species. 11
Supplementary Figure 5. Genome size is not correlated with haploid chromosome number. 12
Supplementary Figure 6. Correspondence between reconstructed ancestral regions with AGORA and Merian elements. 13
Supplementary Figure 7. Correspondence between Merian elements and reconstructed ancestral regions with AGORA. 14
Supplementary Figure 8. Merian elements painted across the chromosomes of *Eupithecia centaureata* demonstrate fission and fusion involving M1 and M6. 15
Supplementary Figure 9. Conservation of gene order in *Lysandra coridon* relative to *Polyommatus icarus* despite numerous fission events. 16
Supplementary Figure 10. Merian elements in two *Tinea* species relative to *Micropterix aruncella*. 17
Supplementary Figure 11. Merian elements in two *Melinaeae* species relative to *Danaus plexippus*. 18

Supplementary Figure 12. Merian elements in *Brenthis ino* relative to *Fabriciana adippe* and Boloria selene. 19
Supplementary Figure 13. Merian elements in *Apeira syringaria* relative to *Selenia dentaria.* 20
Supplementary Figure 14. Merian elements in *Leptidea sinapis* relative to *Anthocharis cardamines*. 21
Supplementary Figure 15. Merian elements in *Operophtera brumata* and *Philereme vetulata* relative to Hydriomena furcata. 22
Supplementary Figure 16. Relationship between repeat density, genome size and chromosome size in *Lysandra*. 23
Supplementary Figure 17. Relationship between repeat density, genome size and chromosome size in *Leptidea*. 24
Supplementary Figure 18. Relationship between repeat density, genome size and chromosome size in *Philereme vetulara* and *Operophtera brumata*. 25
Supplementary Figure 19. Relationship between repeat density, genome size and chromosome size in *Tinea*. 26
Supplementary Figure 20. Relationship between repeat density, genome size and chromosome size in Pierini. 27
Supplementary Figure 21. Relationship between repeat density, genome size and chromosome size in *Apeira*. 28
Supplementary Figure 22. Relationship between repeat density, genome size and chromosome size in *Brenthis*. 29
Supplementary Figure 23. Relationship between repeat density, genome size and chromosome size in *Melinaea*. 30

List of Supplementary Tables

The following Supplementary Tables are too large to be included in the main text or in the Supplementary Information PDF file. They are supplied in one spreadsheet where each tab corresponds to a Supplementary Table.

Supplementary Table 1: Genome assemblies information.
Genome assembly information for the 210 Lepidopteran and 5 Trichopteran genomes used in this study. For each of the genomes analysed, the table provides the assembly name, species, data source (DToL vs INSDC), GCA accession number, assembly level, scaffold N50, percent of assembly scaffolded in chromosomes, BUSCO completeness (lepidoptera odb10) number of duplicate BUSCOs, genome size, chromosome number, taxonomic information and the reference for the assembly.

Supplementary Table 2: W chromosome information.
W chromosome information for each of the 210 lepidopteran genomes. For each genome, the table contains the assembly name, observed sex from the sample sequenced, sex chromosomes identified in the assembly, number of W contigs and size of the W (in Mb) for assemblies where a single contig was assigned as the W.

Supplementary Table 3: Gene annotation information.
Gene annotation information per genome. The table specifies the accession number of each annotated genome, whether the annotation was generated using BRAKER2 or Genebuild, the number of coding genes, the number of mRNA transcripts and the generation date of each annotation is also listed to provide version information.

Supplementary Table 4: Assignment of 4,112 single copy orthologues to Merian elements.
Assignment of 4,112 single-copy orthologues to Merian elements. For each single-copy ortholog, the table specifies which Merian element it was assigned to by syngraph.

Supplementary Table 5: Inferred fusion and fission events with Syngraph.
Fusion and fission events across the 196 lepidopteran species that have undergone simple fusions and/or fissions, inferred using syngraph. For each inferred fusion or fission event, the table contains the parent and child node where the event occurred,

the multiplicity of the event (defined as the number of events), and the Merian elements involved in the event.

Supplementary Table 6: Inferred fusion and fission events with LFFF.

Fusion and fission events across the 196 lepidopteran species which have undergone simple fusions and/or fissions, inferred using Lep Fusion Fission Finder (LFFF). For each inferred fusion or fission event, the table contains the node where the event occurred, the species. That have experienced the event and the Merian elements involved in the event.

Supplementary Table 7: Inferred fusion and fission events with Syngraph for highly rearranged species.

Fusion and fission events inferred with syngraph for highly rearranged lepidopteran species. The table contains the set of fusions and fissions for the 14 species which have either undergone numerous fission events, or complex fusion and fission events. For each inferred fusion or fission event, the table contains the parent and child node where the event occurred, the multiplicity of the event (defined as the number of events) and the Merian elements involved in the event.

Supplementary Table 8: Genomic correlates of chromosome length across 193 species.

Genomic correlates of chromosome length across 193 lepidopteran species. For each species, the table lists the strength of correlation (Spearman's rank) between coding density/ repeat density/ GC3/ synteny/ single-copy orthologues and proportional chromosome length. Spearman's Rank correlation coefficients (R) and p-values were obtained by two-sided Spearman's correlation test are indicated.

Supplementary Table 9: Mean feature density per Merian element across the dataset.

Mean feature density per Merian element across the dataset. The table specifies the mean feature density and standard deviation for a set of features (scaled GC content, scaled GC3 content, scaled repeat density, scaled coding density, synteny, and proportion of single-copy, conserved orthologues relative to multi-copy and singleton genes) per Merian element. Only chromosomes corresponding to intact Merian

elements (i.e. have not undergone fusion or fission) in the 210 lepidopteran genomes were included in these statistics.

Supplementary Table 10: Feature statistics for all 6,254 chromosomes in the dataset.

Feature statistics for all 6,254 chromosomes in the dataset. For each chromosome, the table includes the corresponding species name, assigned Merian elements, rearrangement status, GC,and GC3 values, synteny (%), coding density (%), repeat density (%), the proportion of single copy orthologues (%) chromosome length (bp) and genome size (bp). The table also includes the scaled GC, scaled GC3, scaled repeat density, and scaled proportion of single-copy orthologues. Scaled feature densities were calculated by dividing the value for the chromosome by the scale factor for the genome (the average value for the genome).

Supplementary Table 11: Proportion of each genome annotation by the main TE classifications.

Proportion of each of the 210 lepidopteran genomes annotated by the main TE classifications. Table details the proportion of each lepidopteran genome annotated by the main TE classifications (DNA, LINE, LTR, RC, SINE, Other and Unknown) using Earl Grey. ‘Unknown’ includes repeats classified as unknown, retroposon and unspecified. ‘Other’ includes repeats classified as satellite, simple repeat, and low complexity.

Description of files contained in the Zenodo repository

The following files are publicly available in the Zenodo repository https://doi.org/10.5281/zenodo.7925505 [https://zenodo.org/doi/10.5281/zenodo.7925505][1]:

- Repeat annotations and repeat libraries for each species analysed
- phylogeny_210Leps_5Trichop.treefile - contains the phylogeny inferred using IQ-TREE.
- Files containing Merian elements painted across the chromosomes of each analysed species in PDF format.

Supplementary Text

Section 1. Dataset selection

The genomes used in this study were generated from a mix of female (heterogametic, ZW) and male (homogametic, ZZ) specimens. Therefore, W chromosomes are not present in all assemblies. 61 assemblies were generated from females, of which 27 contained a single scaffold assigned to a W, with mean size of 14.57 Mb (Supplementary Table 2). The remaining assemblies contained 2-84 scaffolds corresponding to the W chromosome. Two assemblies were inferred to be Z0 due to the absence of any W-linked sequence. As the W chromosome is largely composed of repetitive sequence, it was not included in analyses of chromosome structure.

Section 1. Identification of transposable elements

Transposable elements identified with Earl Grey[2] occupy between 67.5% (*Micropterix aruncella*) and 7.4% *(Danaus plexippus*) of each genome with an average of 41.7% (SD=10.8%) (Supplementary Table 10). As previously described for several species, we find that LINEs are the most prevalent transposable element in lepidopteran genomes, followed by rolling circle elements (Supplementary Table 11)[3,4]. Most species show a higher abundance of DNA elements than LTRs and SINEs but SINES are enriched in the genomes of some species.

Section 1. Phylogeny

The dataset used for the phylogeny included a scaffold-level Trichopteran genome (*Hydropsyche tenuis*) in order to increase the taxonomic breadth of Trichoptera used as an outgroup to Lepidoptera. The phylogeny is consistent with previously published time-calibrated phylogenies, including a recent, comprehensive molecular analysis of lepidopteran phylogeny[5]. All 30 families were recovered as monophyletic and *Micropterix aruncella* (Superfamily. Micropterigidae) was positioned as the earliest diverging within Lepidoptera as expected[5].

Section 2. Assignment of orthologues to Merian elements

The assignment of the remaining orthologues to a Merian element or as absent in the last common ancestor will likely be possible with the sequencing of further early-diverging relatives including species of Micropterigoidea, Agathiphagoidea, and Heterobathmioidea. Given that all species that were used to build the lepidoptera odb10 set are from Dytrisia, it is likely that many were simply not present in the last common ancestor of Lepidoptera.

We also inferred the ancestral gene set and gene organisation of Lepidoptera using AGORA46. All orthologues that were assigned to Merian elements by syngraph were also in the ancestral gene set from AGORA. Of the 3093 orthologues that were assigned to Merian elements by syngraph and to contiguous ancestral regions (containing two or more orthologues) by AGORA, we found only a single conflicting orthologue assignment

Section 3. Filtering the dataset when inferring fusion and fission events

Before inferring fusion and fission events, the distribution of Merian elements were visualised across the chromosomes of all 210 lepidopteran genomes in order to check for any data quality issues. This enabled us to be confident that our resulting inferred fusion and fission events were not artefacts due to misassembly. Most genomes were high quality, with only chromosomal scaffolds containing multiple single copy orthologues. However, three genomes contained unlocalised scaffolds with multiple

single copy orthologues which belonged to Merian elements. First, *Spodoptera frugiperda*[6] contained a scaffold (WMCG01000038.1) which contained a set of orthologues corresponding to M5. To prevent this scaffold from being called as a fission event, it was manually removed from the assembly for subsequent analyses. The second, *Dendrolimus kikuchii*[7]*,* contained two scaffolds (JAHHIN010000030.1 and JAHHIN010000032.1) contained 18 and 7 BUSCOs respectively, of which 16 and 6 BUSCOs were duplicated. This suggested that these scaffolds are the result of haplotypic duplication. As their presence prevented a fusion between MZ and M31 from being inferred, these two scaffolds were manually removed from the assembly for subsequent analyses. The third, *Heliconius sara* (GCA 917862395.1) contained four scaffolds which contained sets of orthologues that belonged to Merian elements. Scaffolds CAKJTV010000120.1 and CAKJTV010000131.1 contained 117 and 89 BUSCOs which belong to M17 and M20 respectively. Scaffolds CAKJTV010000302.1 and CAKJTV010000313.1 contained 23 and 9 BUSCOs which belong to M11 and M20 respectively. To prevent these Merian elements from being called as fission events, the assembly was updated by the Darwin Tree of Life team with the above scaffolds assigned to chromosomes. The resulting assembly (GCA_917862395.2) was used in the analysis of fusion and fission events. However, as this updated assembly was not available at the time of analyses of gene and repeat content, it was not used in these analyses.

Supplementary Figures

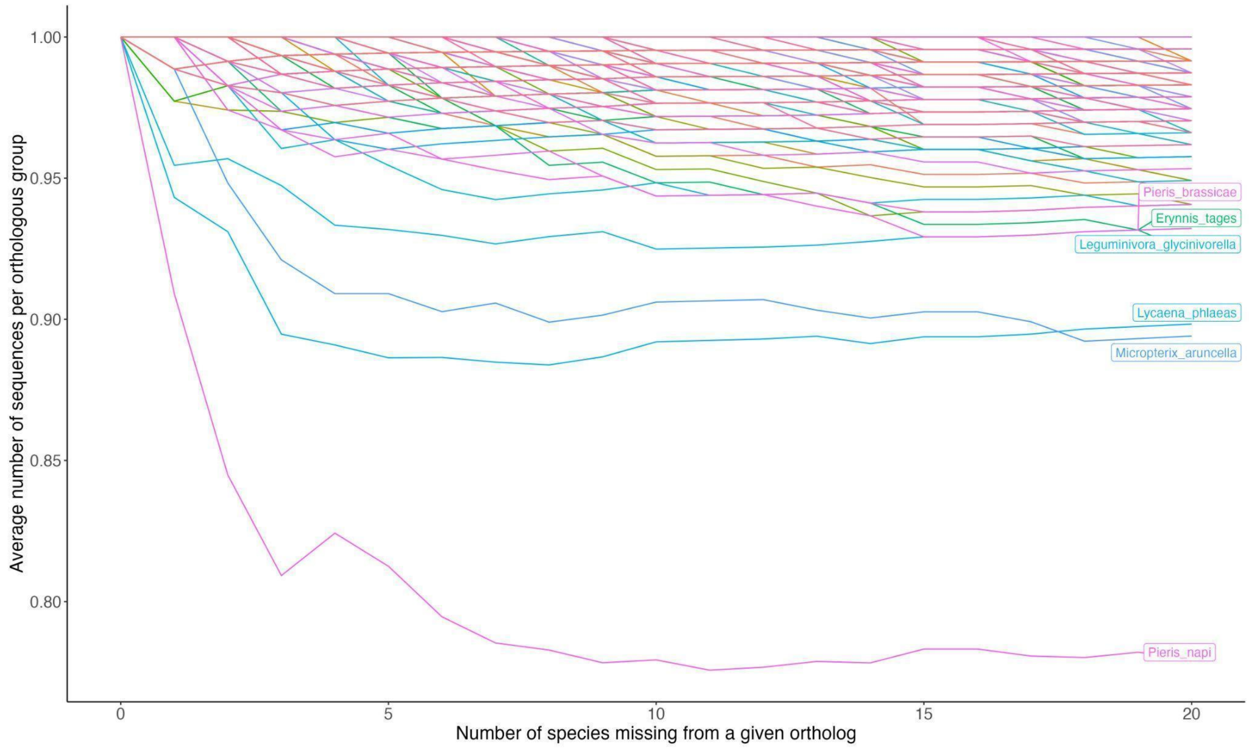

Supplementary Figure 1. Assessing quality of gene annotations using the number of missing conserved single-copy genes.

The average number of sequences per orthologous group. We varied the number of species allowed to be missing from an orthologous group that was otherwise single-copy and conserved in all other species. Species with a low average number of sequences per orthologous group, such as *Pieris napi,* have relatively incomplete gene sets and/or genomes.

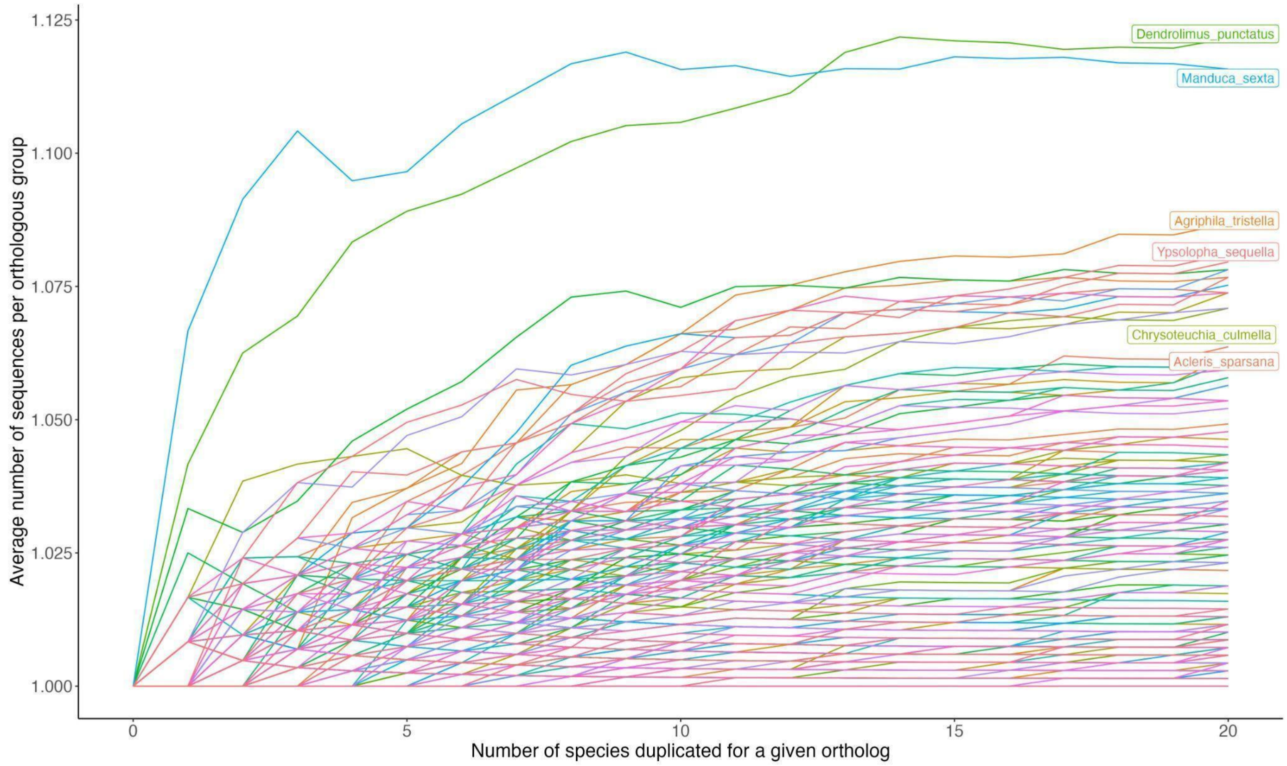

Supplementary Figure 2. Assessing quality of gene annotations using the number of duplicated conserved single-copy genes.

The average number of sequences per orthologous group. We varied the number of species allowed to have two sequences per an orthologous group that was otherwise single-copy and conserved in all other species. Species with a high average number of sequences per orthologous group, such as *Dendrolimus punctutus and Manduca sexta*, have relatively duplicated gene sets and/or genomes.

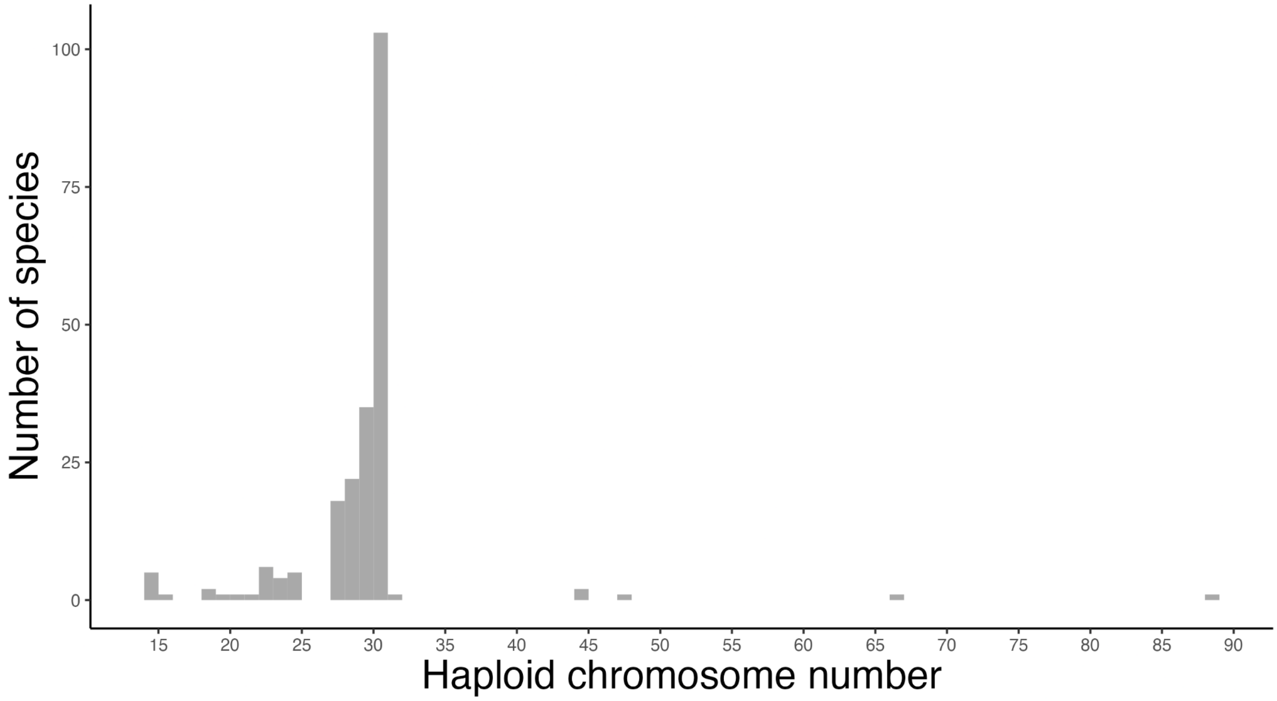

Supplementary Figure 3. Distribution of haploid chromosome number in Lepidoptera from 210 species.
Chromosome numbers obtained from chromosomal genome sequences, including the Z but not W chromosome(s).

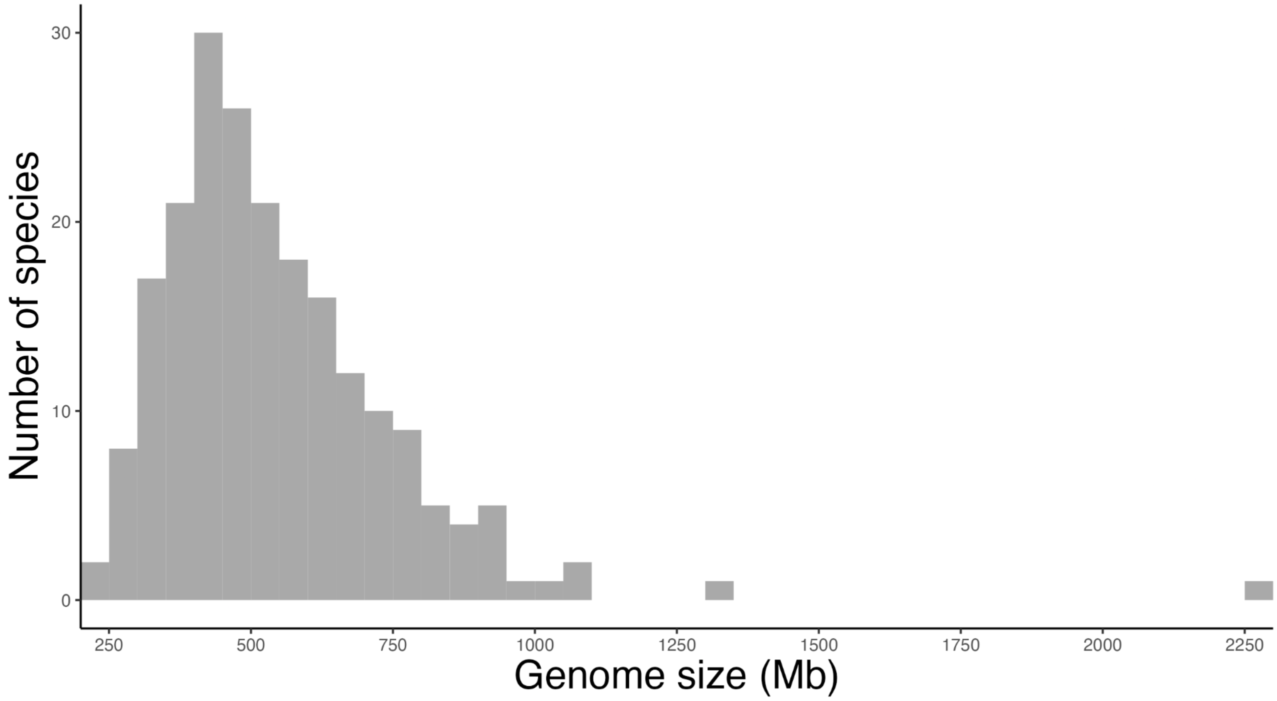

Supplementary Figure 4. Distribution of genome size (Mb) in Lepidoptera from 210 species.
Genome sizes obtained from chromosomal genome sequences.

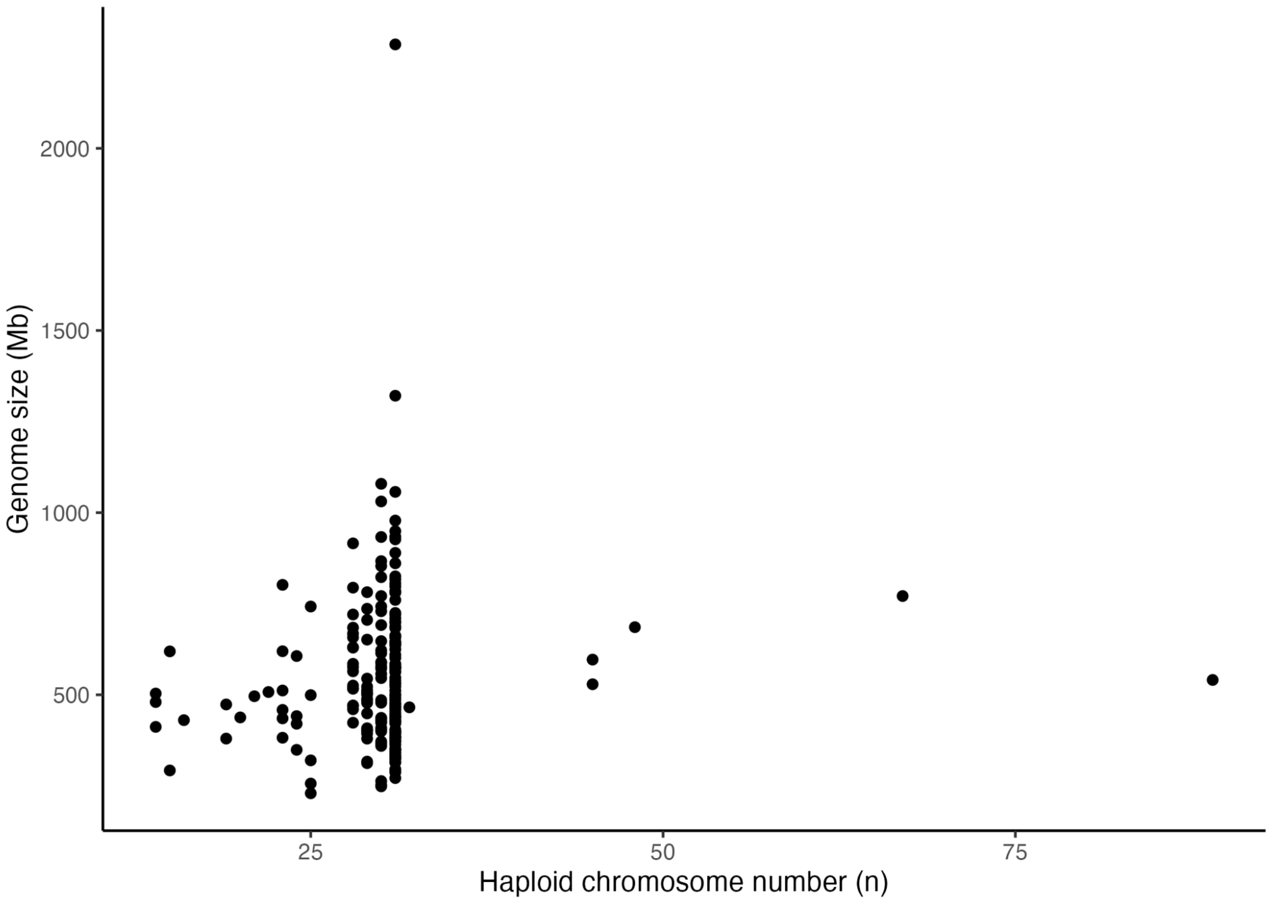

Supplementary Figure 5. Genome size is not correlated with haploid chromosome number.

To account for shared ancestry between species, a phylogenetic linear model was constructed with genome size as the response variable and chromosome number as the predictor variable . The most appropriate model for the error terms was identified as Ornstein-Uhlenbeck (OU) based on comparison of AIC values. The resulting phylogenetic linear model found no significant correlation between genome size and haploid chromosome number (t=0.83, p=0.4087, adjusted r2 = 0.00795). Genome size and chromosome numbers obtained from 210 chromosomal genome sequences.

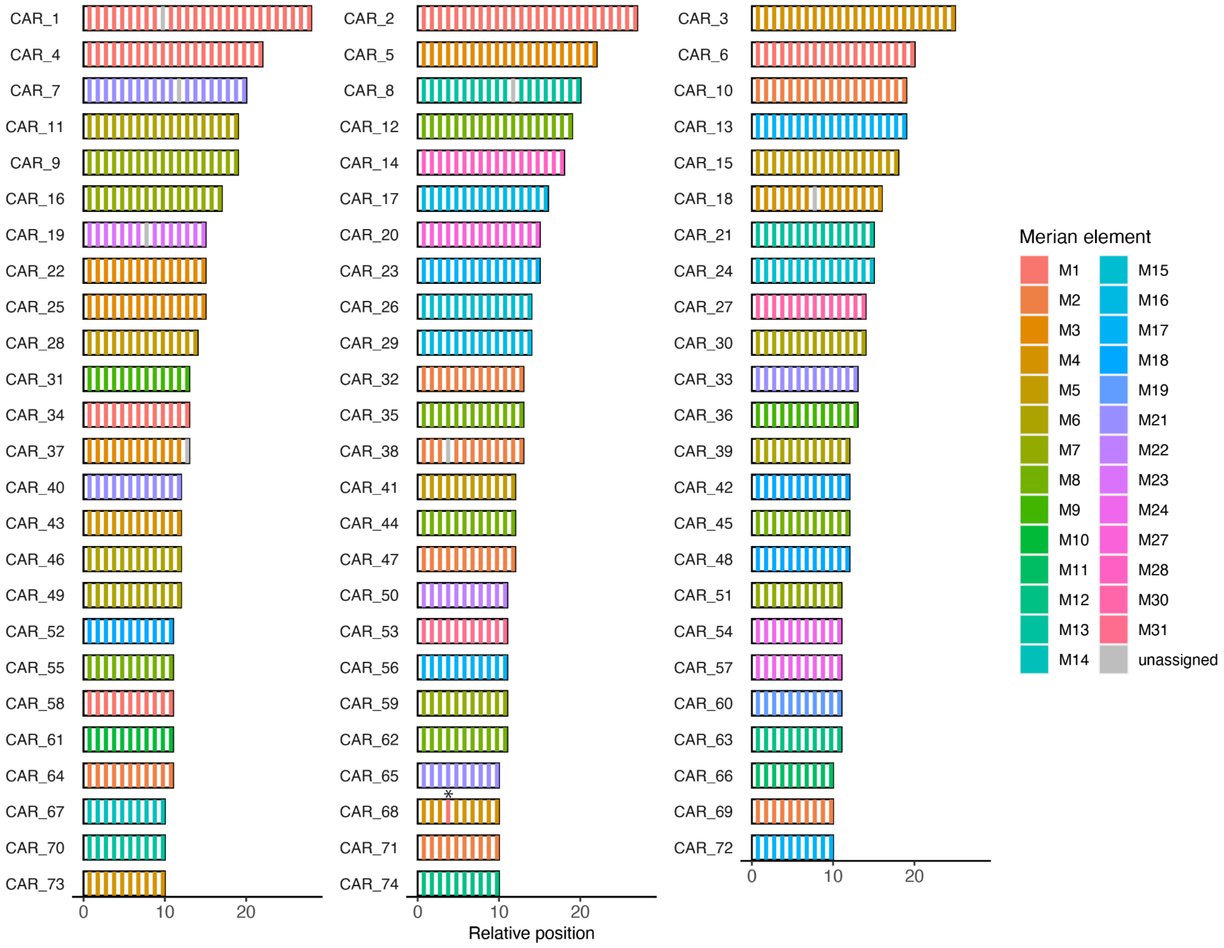

Supplementary Figure 6. Correspondence between reconstructed ancestral regions with AGORA and Merian elements.

Merian elements painted across the contiguous ancestral regions (CARs) of the last common ancestor of *M. aruncella* and *T. trinotella* using AGORA. Each reconstructed region is represented by a rectangle within which the position of each ortholog is painted the colour of the Merian element it is assigned to. All CARs with at least 10 orthologues are plotted. Each CAR contained orthologues corresponding to a single Merian element with the exception of CAR_68 which contained a single conflicting orthologue which mapped to M1 rather than M4 which all other orthologues map to (indicated by the asterix).

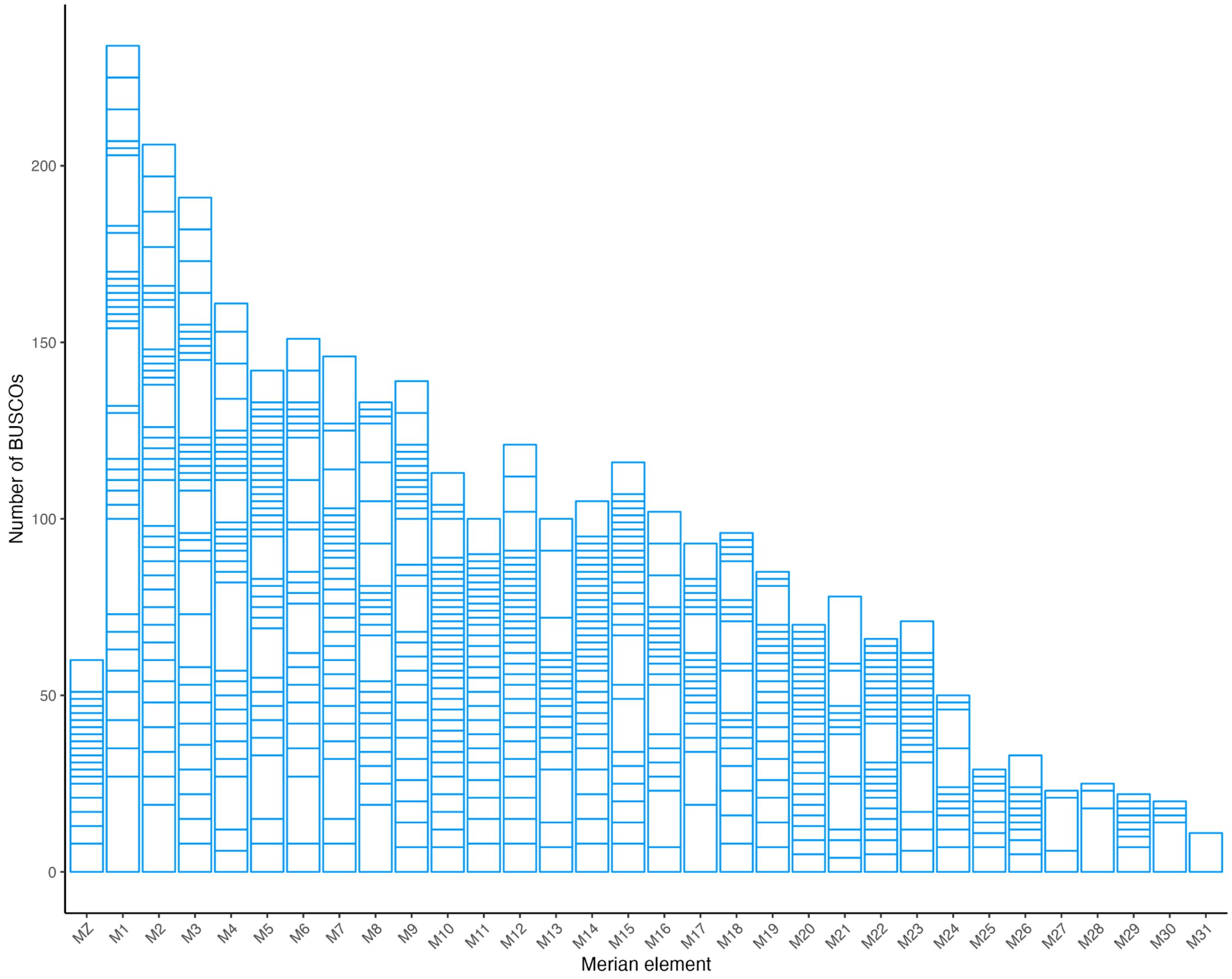

Supplementary Figure 7. Correspondence between Merian elements and reconstructed ancestral regions with AGORA.

Stacked bar chart of the number of Merian-defining orthologues per contiguous ancestral region (CAR) for Lepidoptera inferred from AGORA per Merian element. The orthologue content of each CAR corresponds to a single Merian element with the exception of a single CAR: CAR_68 contains one orthologue derived from M1, whereas the remaining 9 orthologous correspond to M4.

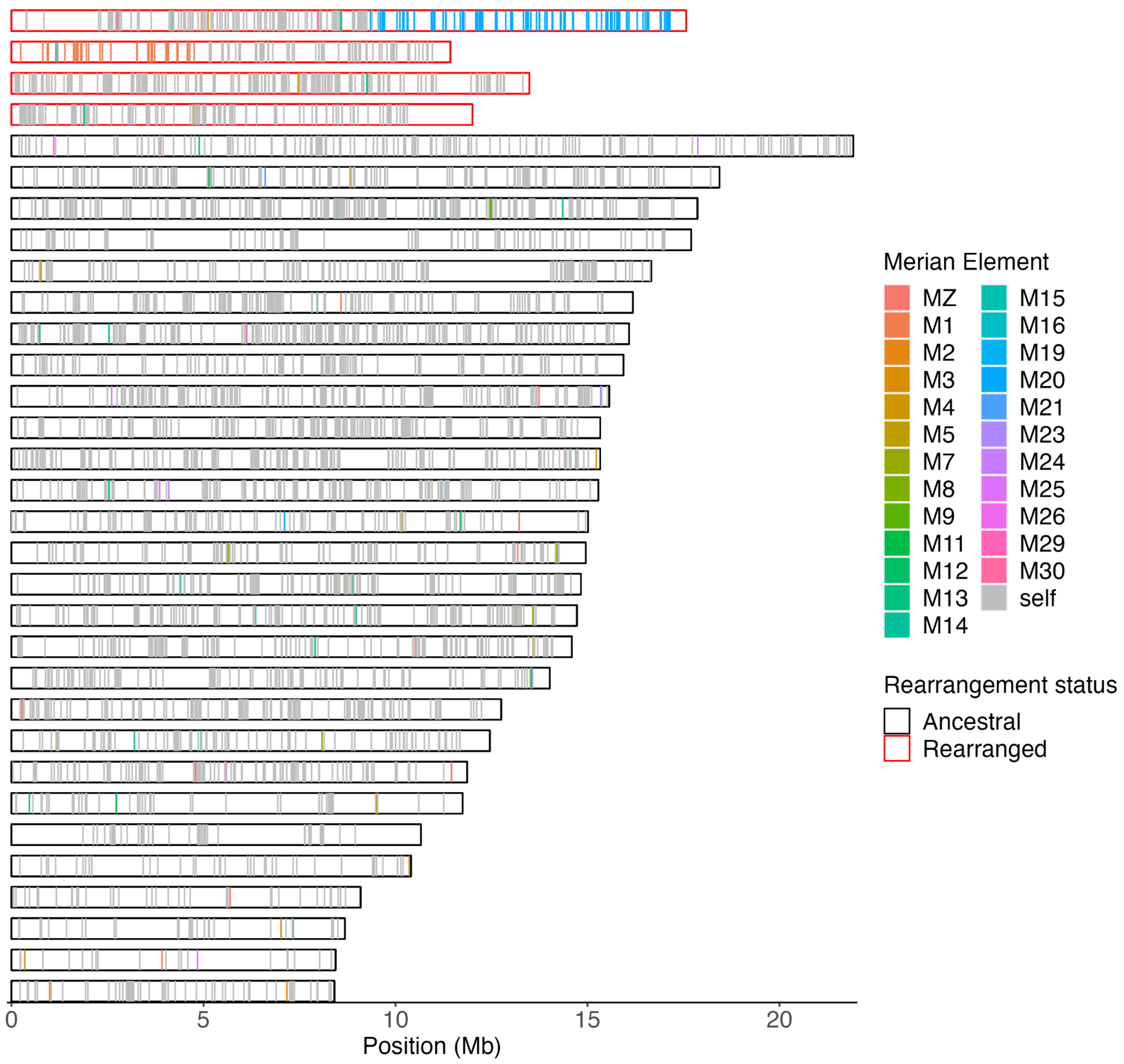

Supplementary Figure 8. Merian elements painted across the chromosomes of *Eupithecia centaureata* demonstrate fission and fusion involving M1 and M6. Each chromosome is represented by a rectangle within which the position of each ortholog is grey if it belongs to the most common Merian element for that chromosome or is coloured if it belongs to an alternative Merian element. Chromosomes that have undergone fusions and/or fission events are outlined in red. This reveals a segment of M1 has fused to a segment of M6 (row 2). The remainder of M1 and the remainder of M6 exist as two separate chromosomes (row 3 and 4 respectively), indicating two fission events.

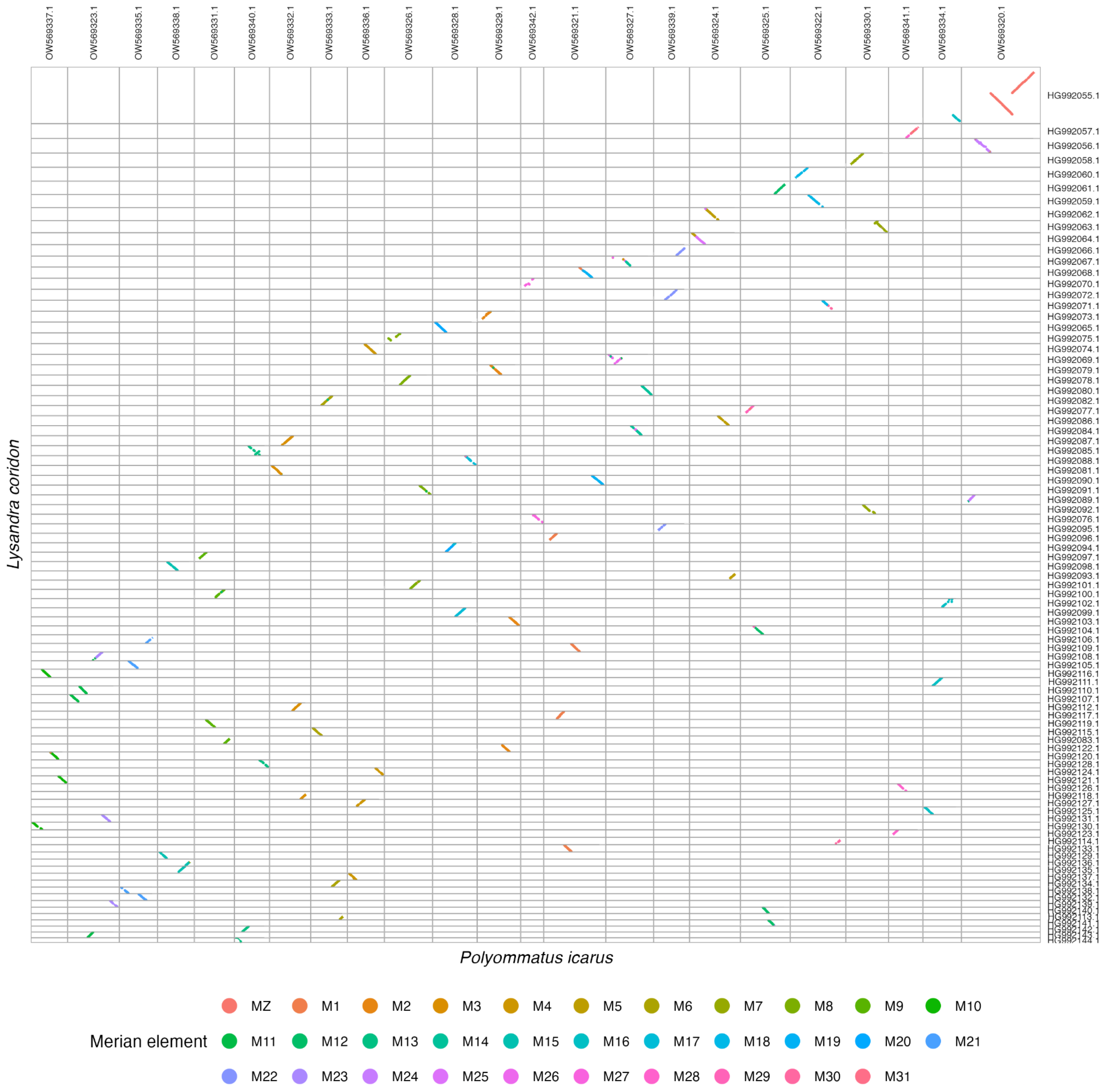

Supplementary Figure 9. Conservation of gene order in *Lysandra coridon* relative to *Polyommatus icarus* despite numerous fission events.

Oxford plot of relative ortholog positions in the genomes of *Lysandra coridon* and *Polyommatus icarus.* Orthologues are coloured by Merian element.

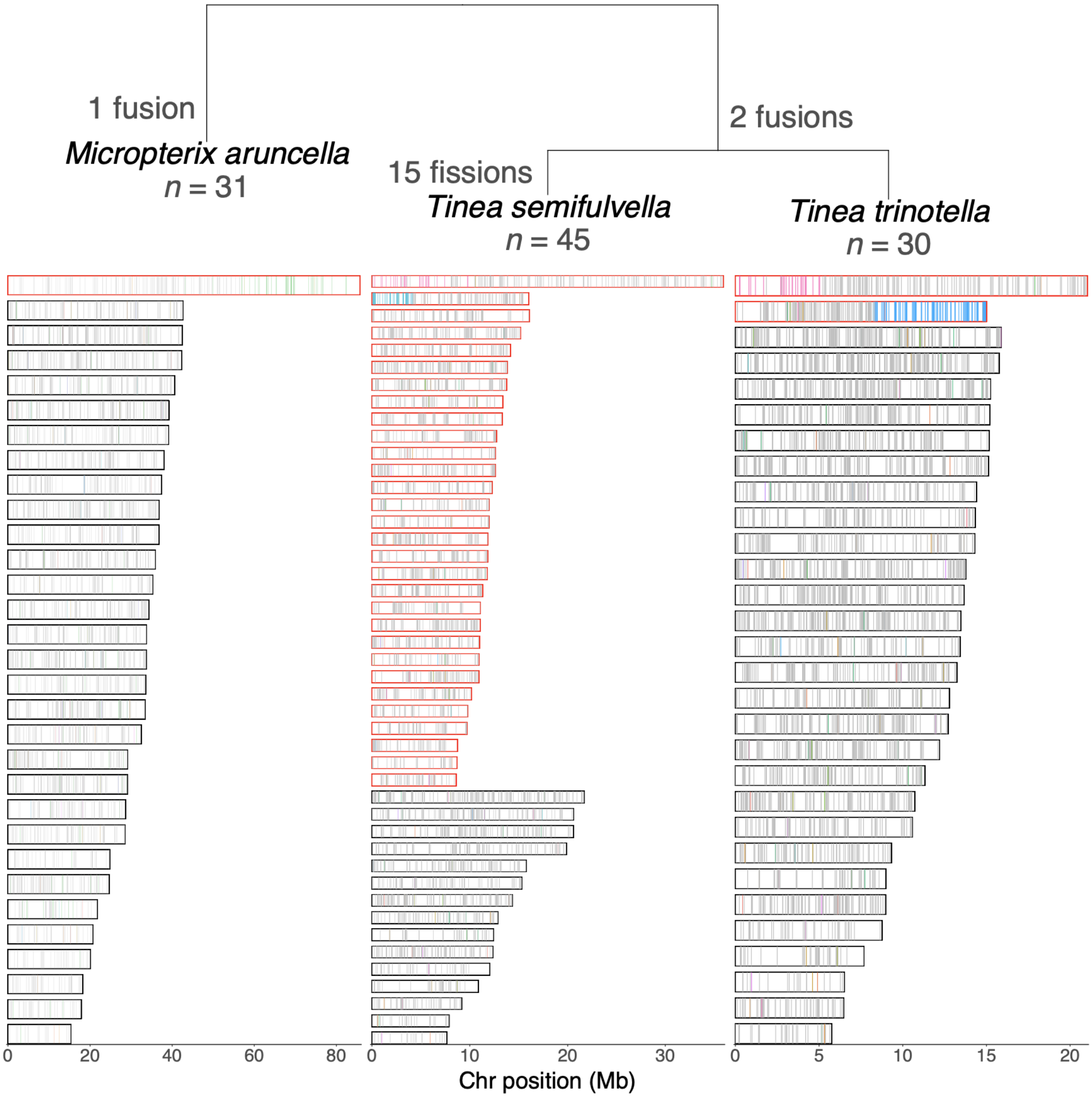

Supplementary Figure 10. Merian elements in two *Tinea* species relative to *Micropterix aruncella.*

Relationship between *Tinea semifulvella* and *T. trinotella* and *Micropterix aruncella* annotated with inferred fusion and fission events at each node.

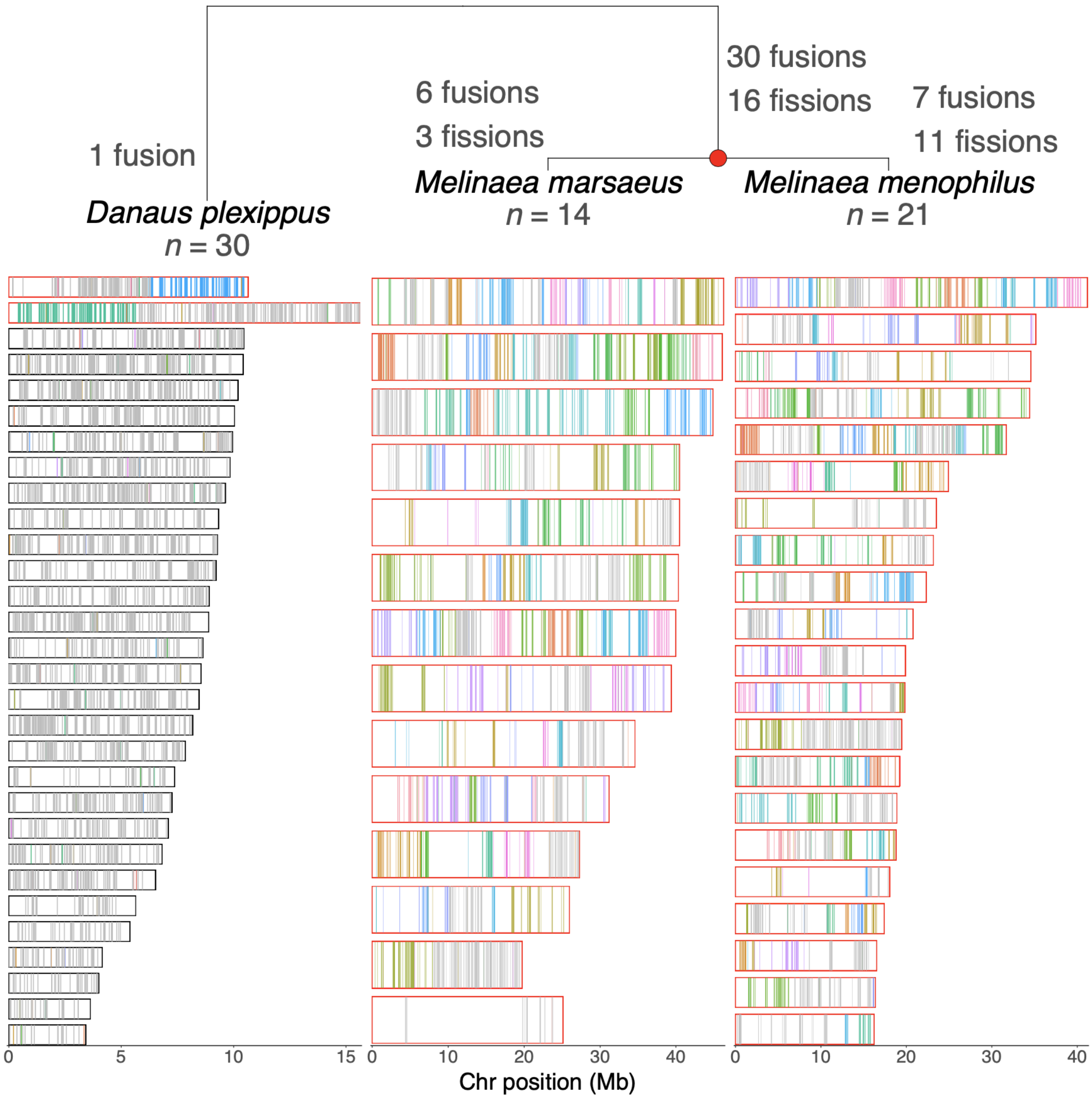

Supplementary Figure 11. Merian elements in two *Melinaeae* species relative to *Danaus plexippus.*

Relationship between *Melinaea marsaeus, M. menophilus* and *Danaus plexippus* annotated with inferred fusion and fission events at each node.

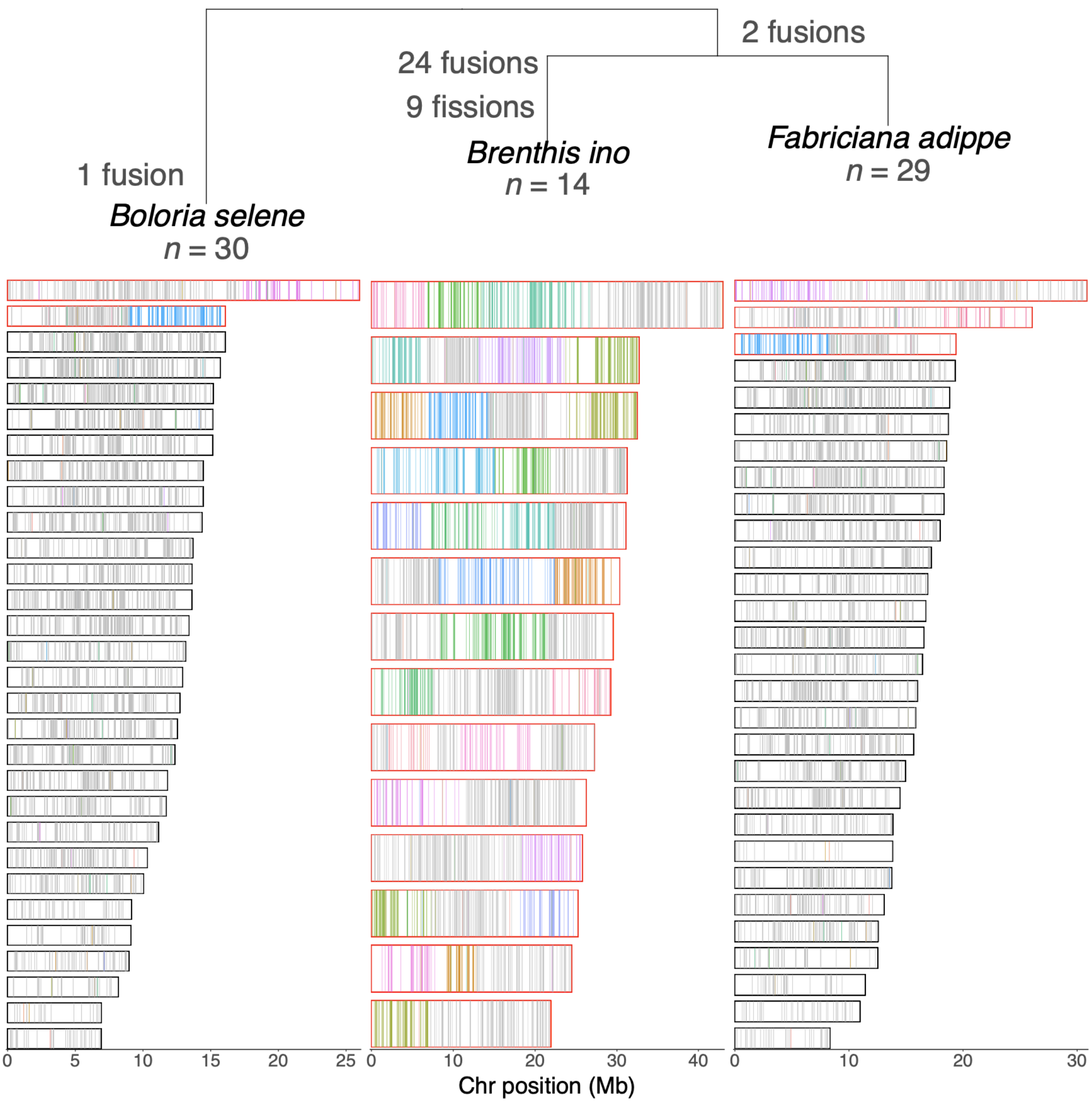

Supplementary Figure 12. Merian elements in *Brenthis ino* relative to *Fabriciana adippe* and *Boloria selene.*

Relationships between *Brenthis ino, Fabriciana adippe*a and *Boloria selene* annotated with inferred fusion and fission events at each node.

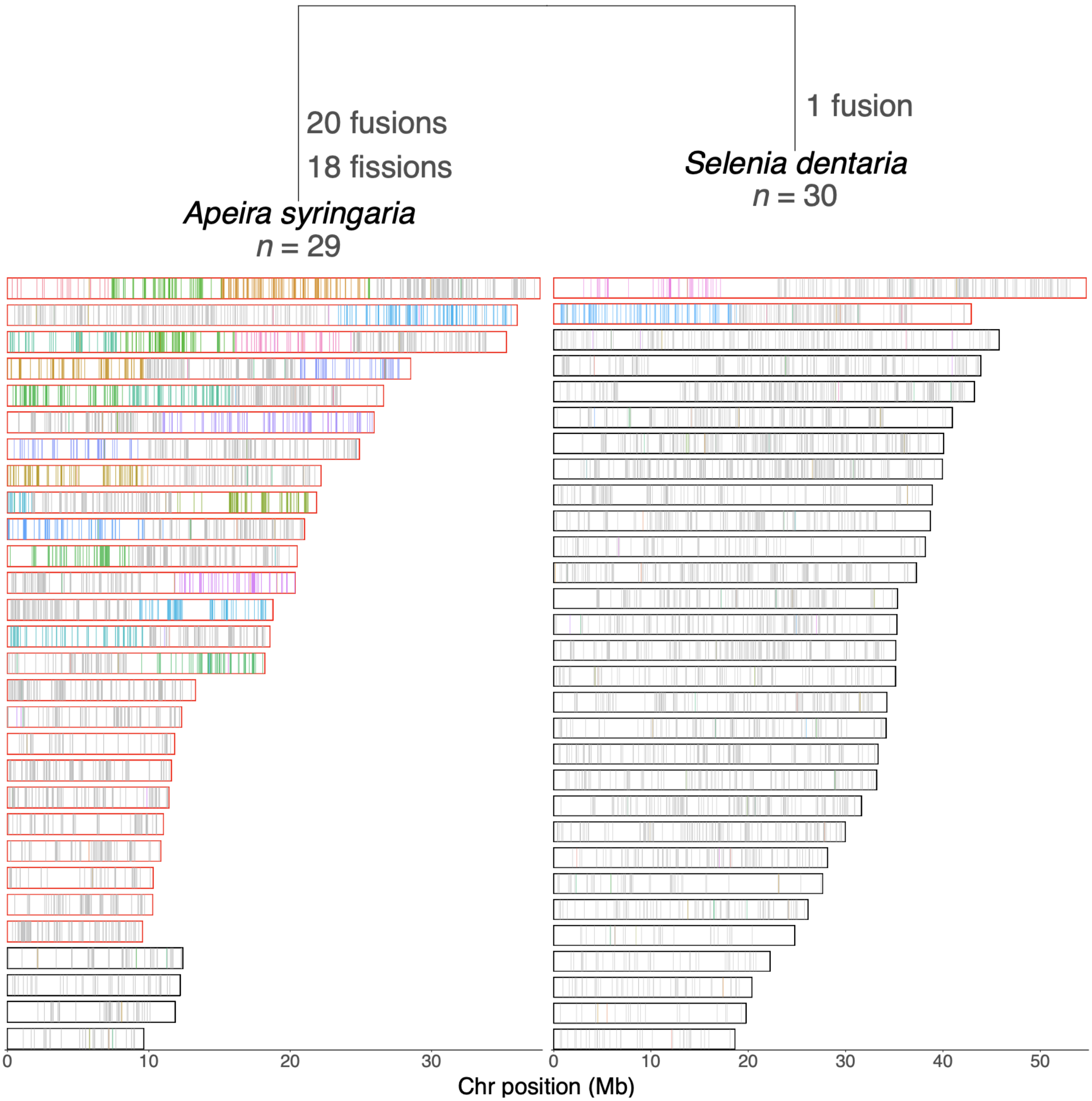

Supplementary Figure 13. Merian elements in *Apeira syringaria* relative to *Selenia dentaria.*

Relationship between *Apeira syringaria* and *Selenia dentaria* annotated with inferred fusion and fission events at each node.

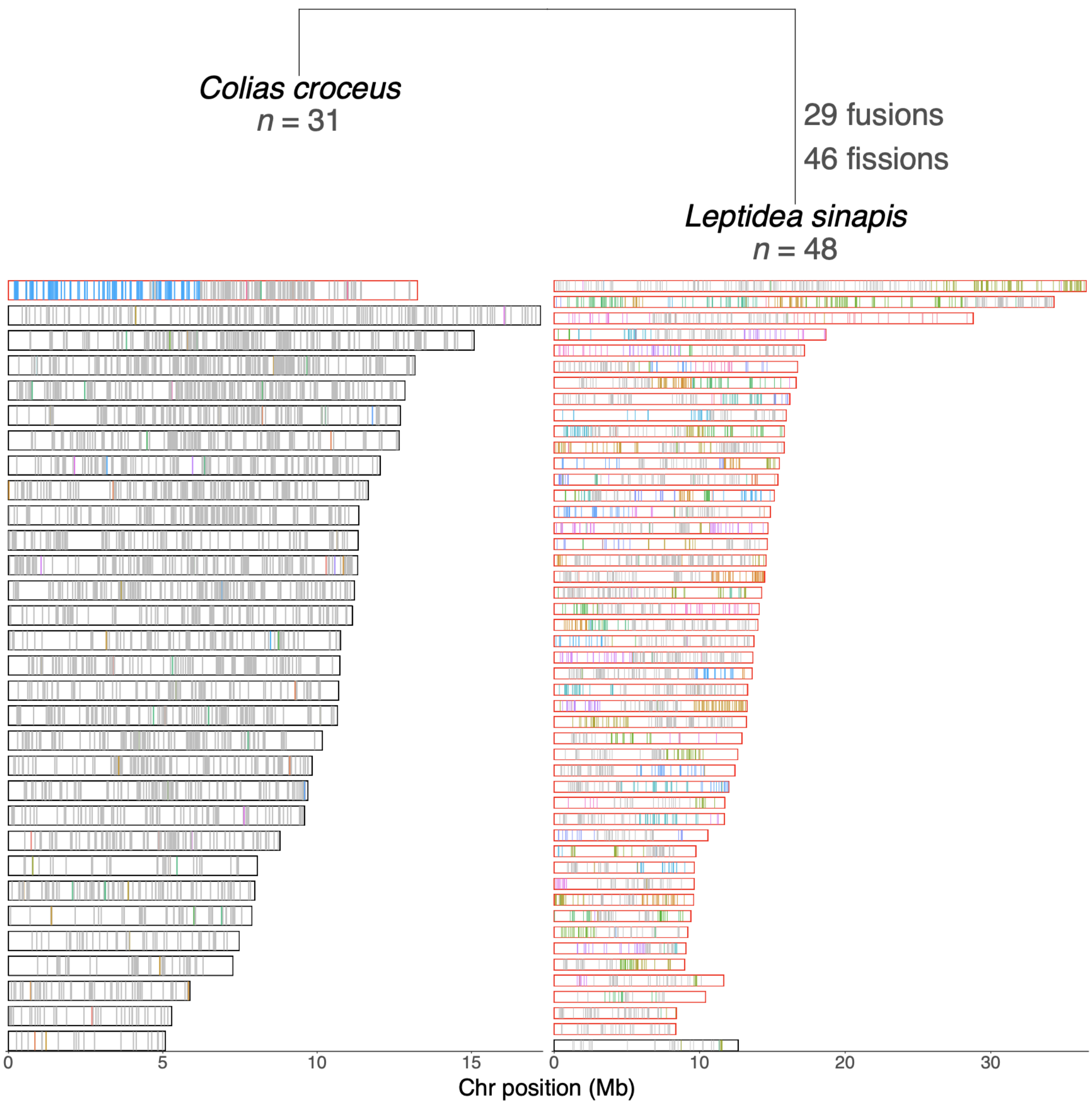

Supplementary Figure 14. Merian elements in *Leptidea sinapis* relative to *Anthocharis cardamines.*

Relationships between *Colias croceus* and *Leptidea sinapis* annotated with inferred fusion and fission events at each node.

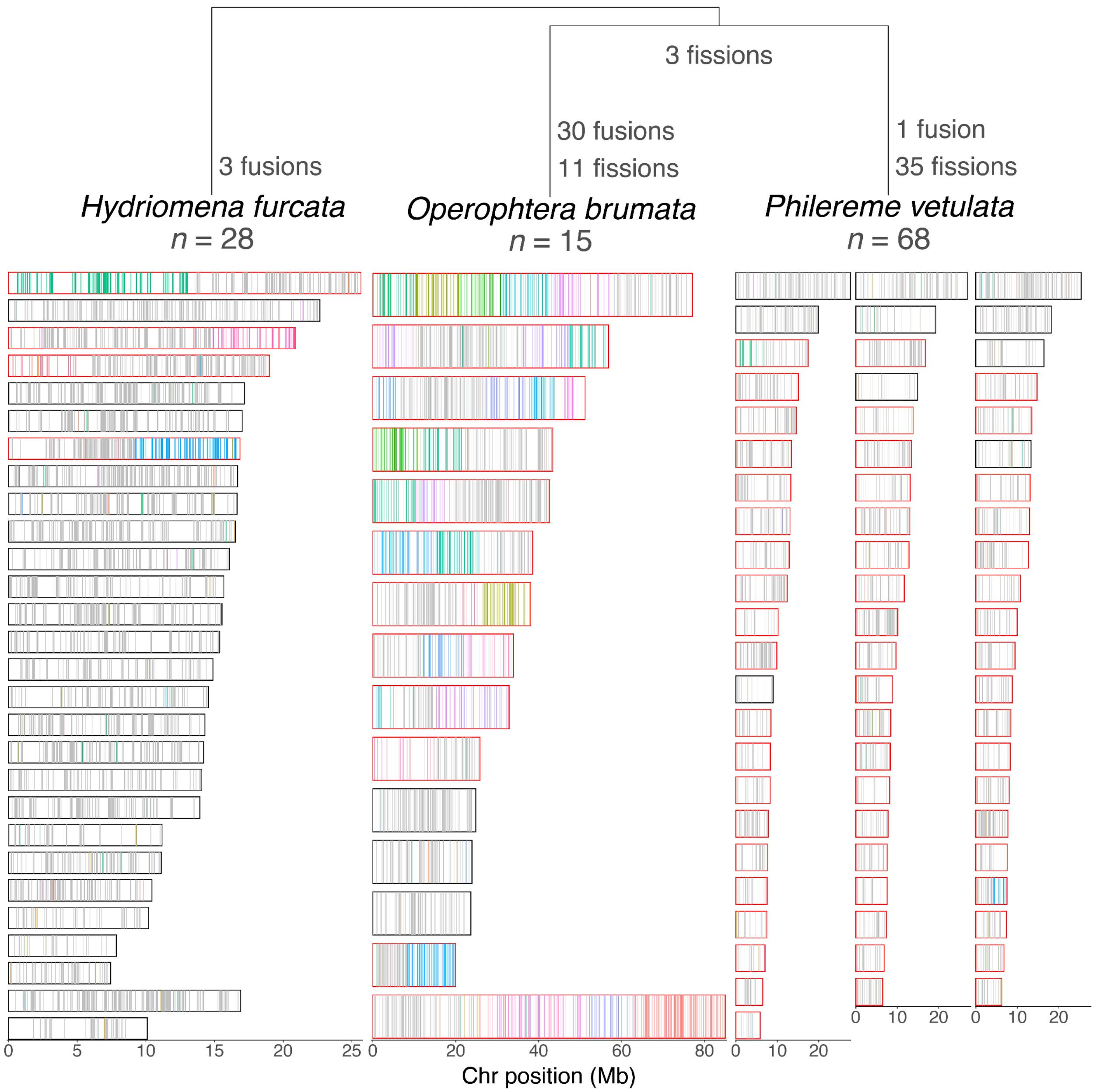

Supplementary Figure 15. Merian elements in *Operophtera brumata* and *Philereme vetulata* relative to *Hydriomena furcata.*
Relationships between *Hydriomena furcata*, *Operophtera brumata* and *Philereme vetulata* annotated with inferred fusion and fission events at each node.

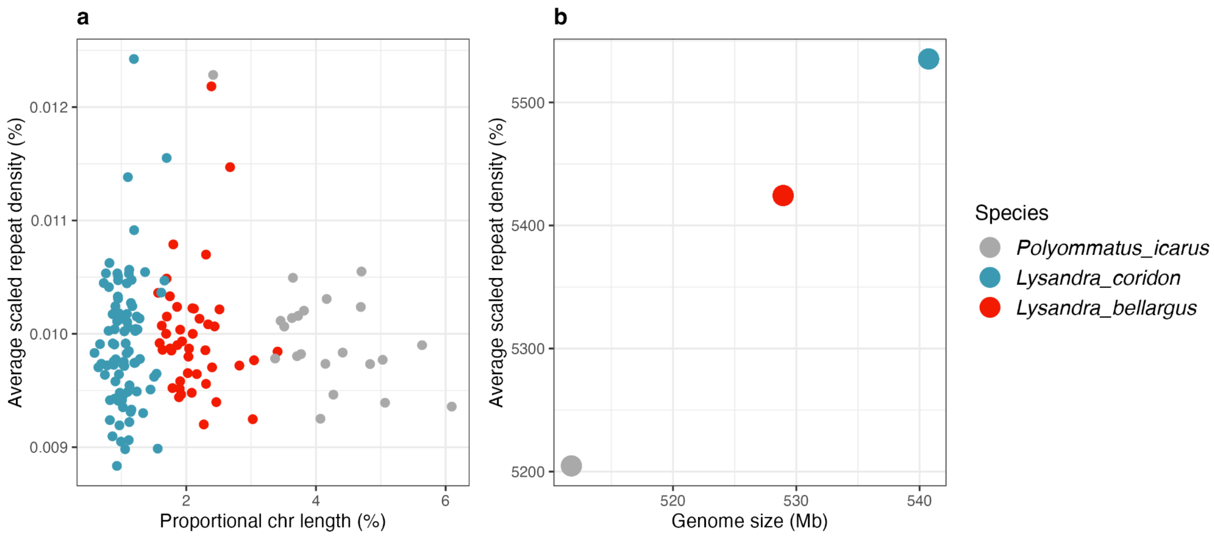

Supplementary Figure 16. Relationship between repeat density, genome size and chromosome size in *Lysandra*.

a, b, Mean scaled repeat density (%) compared to proportional chromosome length (%) **(a)** and genome size (Mb) **(b)**.

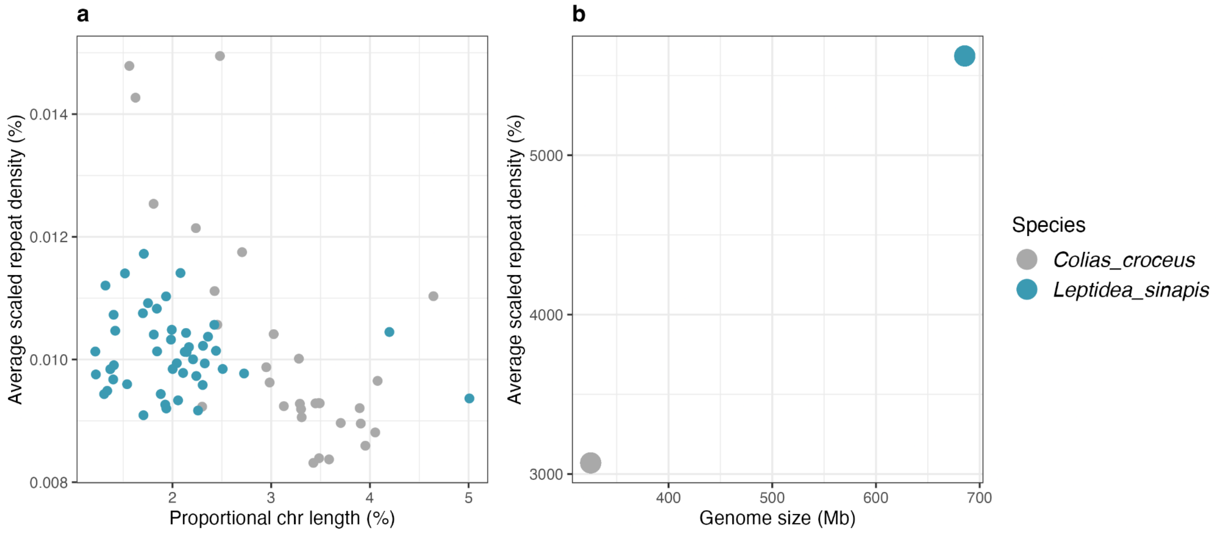

Supplementary Figure 17. Relationship between repeat density, genome size and chromosome size in *Leptidea.*

a, b, Mean scaled repeat density (%) compared to proportional chromosome length (%) **(a)** and genome size (Mb) **(b)**.

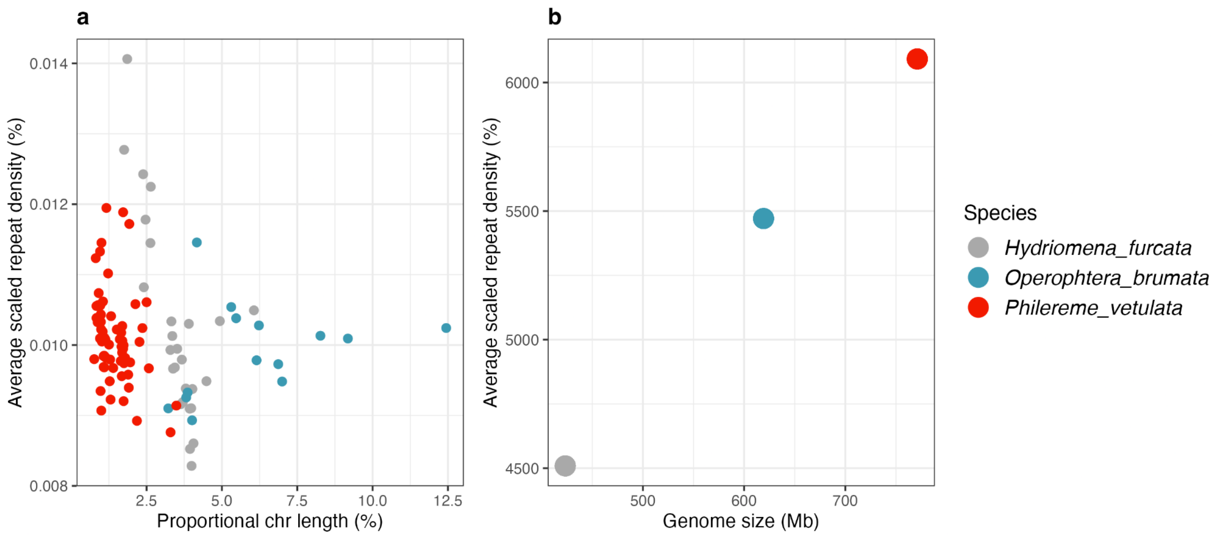

Supplementary Figure 18. Relationship between repeat density, genome size and chromosome size in *Philereme vetulara* and *Operophtera brumata.*

a, b, Mean scaled repeat density (%) compared to proportional chromosome length (%) **(a)** and genome size (Mb) **(b)**.

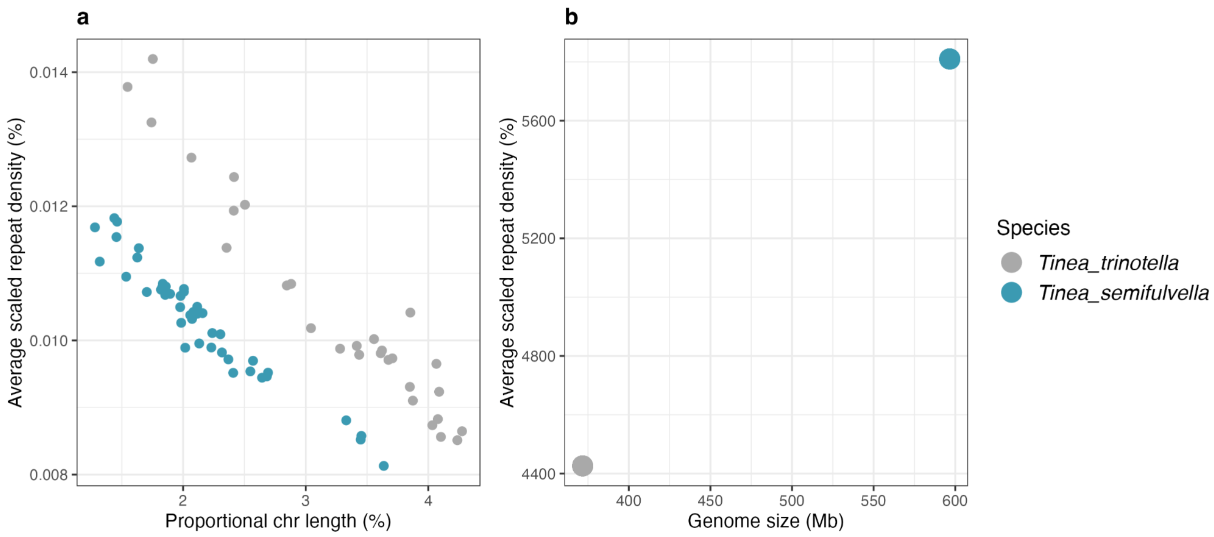

Supplementary Figure 19. Relationship between repeat density, genome size and chromosome size in *Tinea.*

Mean scaled repeat density (%) compared to proportional chromosome length (%) (a) and genome size (Mb) (b).

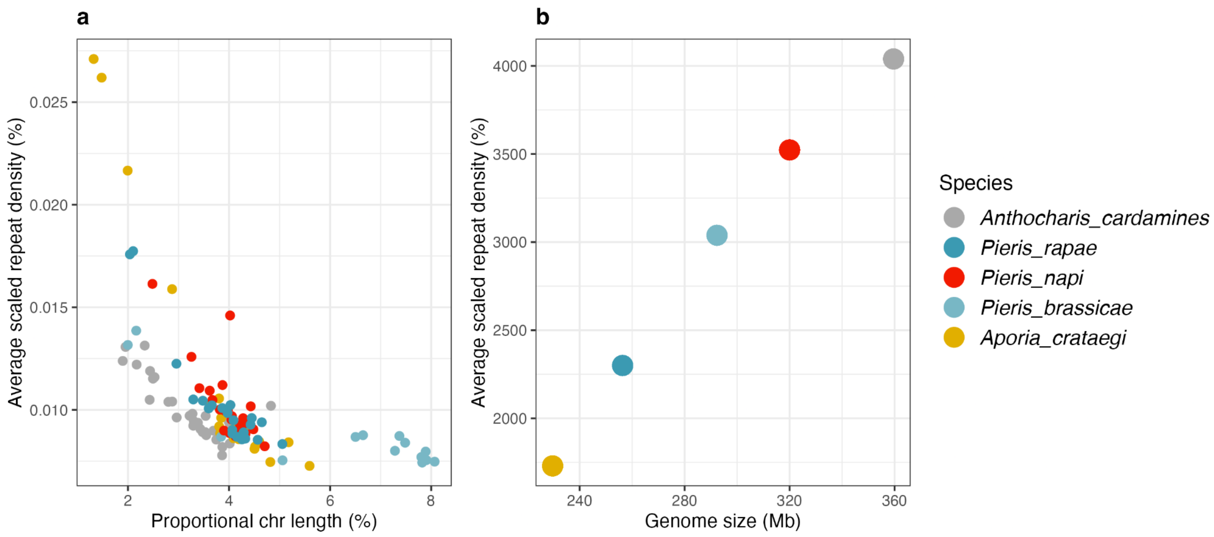

Supplementary Figure 20. Relationship between repeat density, genome size and chromosome size in Pierini.

Mean scaled repeat density (%) compared to proportional chromosome length (%) **(a)** and genome size (Mb) **(b)**.

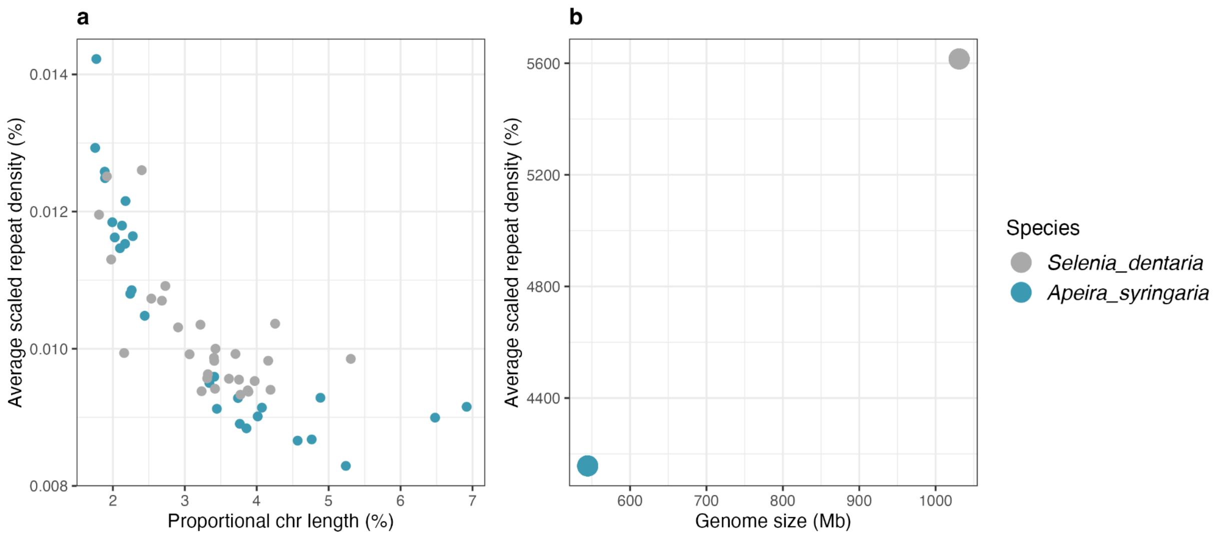

Supplementary Figure 21. Relationship between repeat density, genome size and chromosome size in *Apeira.*

Mean scaled repeat density (%) compared to proportional chromosome length (%) **(a)** and genome size (Mb) **(b)**.

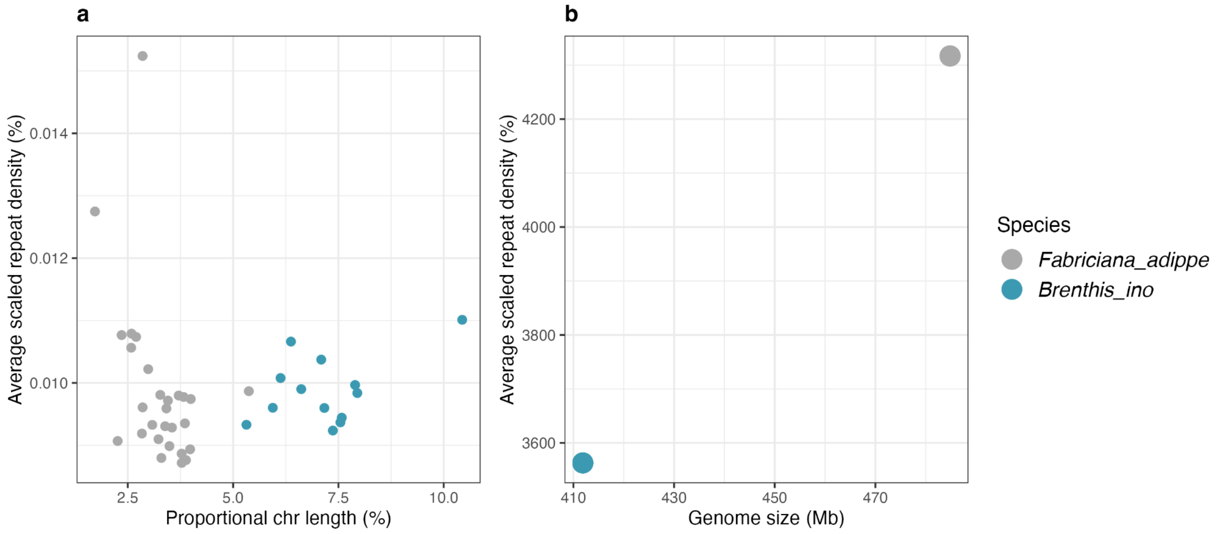

Supplementary Figure 22. Relationship between repeat density, genome size and chromosome size in *Brenthis.*

Mean scaled repeat density (%) compared to proportional chromosome length (%) **(a)** and genome size (Mb) **(b).**

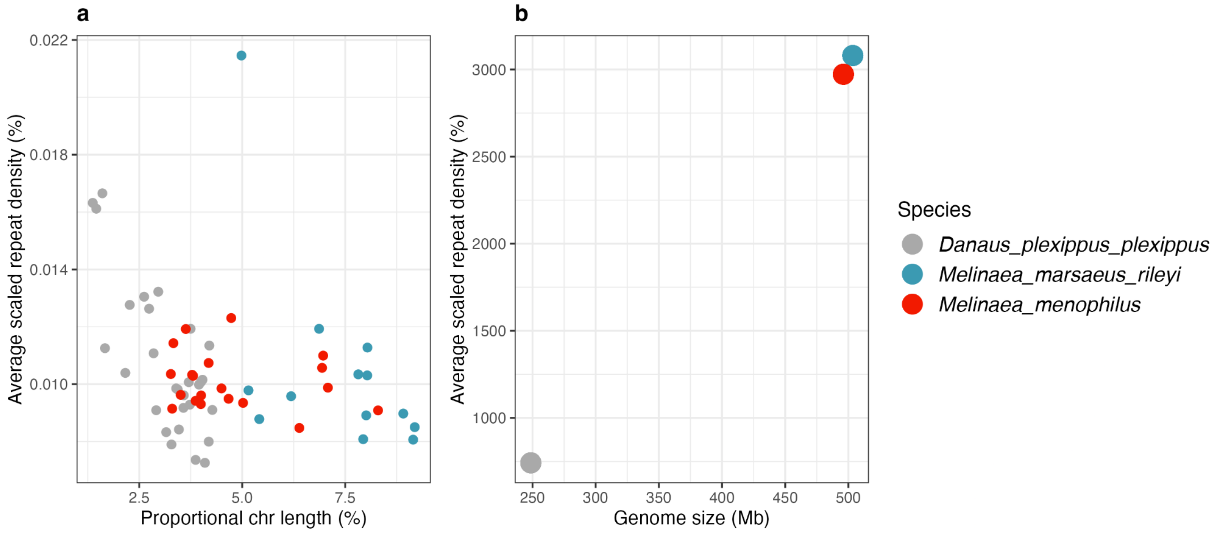

Supplementary Figure 23. Relationship between repeat density, genome size and chromosome size in *Melinaea.*

Mean scaled repeat density (%) compared to proportional chromosome length (%) **(a)** and genome size (Mb) **(b)**.

1. Wright, C. J. Chromosome evolution in Lepidoptera. (2023) doi:10.5281/zenodo.7925505.
2. Baril, T., Imrie, R. M. & Hayward, A. Earl Grey: a fully automated user-friendly transposable element annotation and analysis pipeline. (2022) doi:10.21203/rs.3.rs-1812599/v1.
3. Baril, T. & Hayward, A. Migrators within migrators: exploring transposable element dynamics in the monarch butterfly, Danaus plexippus. *Mob. DNA* **13**, 5 (2022).
4. Mackintosh, A. *et al.* The genome sequence of the scarce swallowtail, Iphiclides podalirius. *G3* **12**, (2022).
5. Kawahara, A. Y. *et al.* Phylogenomics reveals the evolutionary timing and pattern of butterflies and moths. *Proc. Natl. Acad. Sci. U. S. A.* **116**, 22657–22663 (2019).
6. Xiao, H. *et al.* The genetic adaptations of fall armyworm Spodoptera frugiperda facilitated its rapid global dispersal and invasion. *Mol. Ecol. Resour.* **20**, 1050–1068 (2020).
7. Zhou, J. *et al.* Chromosome-Level Genome Assembly Reveals Significant Gene Expansion in the Toll and IMD Signaling Pathways of Dendrolimus kikuchii. *Front. Genet.* **12**, 728418 (2021).
