# Supplementary material for: Comparative genomics reveals the dynamics of chromosome evolution in Lepidoptera
Source: Nat Ecol Evol. 2024 Feb 21;8(4):777–90. doi: 10.1038/s41559-024-02329-4 (PMC11009112; doi:10.1038/s41559-024-02329-4)
Supplement: Supplementary file 2 — Reporting Summary [file 41559_2024_2329_MOESM2_ESM.pdf]

Corresponding author(s): Charlotte J. Wright, Mark Blaxter

Last updated by author(s): Oct 4, 2023

Reporting Summary

Nature Portfolio wishes to improve the reproducibility of the work that we publish. This form provides structure for consistency and transparency in reporting. For further information on Nature Portfolio policies, see our Editorial Policies and the Editorial Policy Checklist.

Statistics

For all statistical analyses, confirm that the following items are present in the figure legend, table legend, main text, or Methods section.

n/a	Confirmed	
☐	☒	The exact sample size (*n*) for each experimental group/condition, given as a discrete number and unit of measurement
☐	☒	A statement on whether measurements were taken from distinct samples or whether the same sample was measured repeatedly
☐	☒	The statistical test(s) used AND whether they are one- or two-sided<br>*Only common tests should be described solely by name; describe more complex techniques in the Methods section.*
☐	☒	A description of all covariates tested
☒	☐	A description of any assumptions or corrections, such as tests of normality and adjustment for multiple comparisons
☐	☒	A full description of the statistical parameters including central tendency (e.g. means) or other basic estimates (e.g. regression coefficient) AND variation (e.g. standard deviation) or associated estimates of uncertainty (e.g. confidence intervals)
☐	☒	For null hypothesis testing, the test statistic (e.g. *F*, *t*, *r*) with confidence intervals, effect sizes, degrees of freedom and *P* value noted<br>*Give P values as exact values whenever suitable.*
☒	☐	For Bayesian analysis, information on the choice of priors and Markov chain Monte Carlo settings
☐	☒	For hierarchical and complex designs, identification of the appropriate level for tests and full reporting of outcomes
☐	☒	Estimates of effect sizes (e.g. Cohen's *d*, Pearson's *r*), indicating how they were calculated

Our web collection on statistics for biologists contains articles on many of the points above.

Software and code

Policy information about availability of computer code

Data collection

No software was used to collect data.

Data analysis

All custom code developed for this manuscript is available at https://github.com/charlottewright/Chromosome_evolution_Lepidoptera_MS which has been accessioned in the Zenodo repository https://doi.org/10.5281/zenodo.10373060.
All third-party software used in the manuscript, including versions, is detailed in the methods along with relevant citations and listed below.

Third-party software and versions used in the manuscript:
Earl Grey (v1.2)
BUSCO (v.5.4.3)
MAFFT (v7.475)
trimal (v1.4)
IQ-TREE (v2.03)
phylolm R package (v2.6.2)
BEDtools (v2.30.0)
AGAT (v1.0.0)
samtools (v1.7)
OrthoFinder (v2.5.4).
Phylolm (2.6.2)
ete3 (3.1.3)
stats R package (4.1.0)
fasta_windows (0.2.4)

The following software tools are not versioned but are available on GitHub, as indicated in the methods section of the manuscript: busco2fasta, catfasta2phyml, gff-stats, genomics_tools, syngraph, lep_fusion_fission_finder, lep_busco_painter, assemblage.

For manuscripts utilizing custom algorithms or software that are central to the research but not yet described in published literature, software must be made available to editors and reviewers. We strongly encourage code deposition in a community repository (e.g. GitHub). See the Nature Portfolio guidelines for submitting code & software for further information.

Data

Policy information about availability of data

All manuscripts must include a data availability statement. This statement should provide the following information, where applicable:

- Accession codes, unique identifiers, or web links for publicly available datasets
- A description of any restrictions on data availability
- For clinical datasets or third party data, please ensure that the statement adheres to our policy

The reference genomes analysed in this study are available at https://www.ncbi.nlm.nih.gov/ and the accession numbers are given in Supplementary Table 1. Gene annotations are available at Rapid.ensembl.org and are listed in Supplementary Table 3. The Arthropoda library from Dfam release 3.5 used to identify transposable elements is available at https://www.dfam.org/releases/Dfam_3.5. Large data files associated with this manuscript, including repeat annotations, repeat libraries and phylogenies are available in the https://doi.org/10.5281/zenodo.7925505 [https://zenodo.org/doi/10.5281/zenodo.7925505]. Other data that supports the findings presented in this paper are available in the Supplementary Tables and on GitHub https://github.com/charlottewright/Chromosome_evolution_Lepidoptera_MS which has been accessioned in Zenodo at https://doi.org/10.5281/zenodo.10373060. Additional source data associated with the figures and tables can be found in the Source Data file.

Research involving human participants, their data, or biological material

Policy information about studies with human participants or human data. See also policy information about sex, gender (identity/presentation), and sexual orientation and race, ethnicity and racism.

Reporting on sex and gender	N/A
Reporting on race, ethnicity, or other socially relevant groupings	N/A
Population characteristics	N/A
Recruitment	N/A
Ethics oversight	N/A

Note that full information on the approval of the study protocol must also be provided in the manuscript.

Field-specific reporting

Please select the one below that is the best fit for your research. If you are not sure, read the appropriate sections before making your selection.

☒ Life sciences ☐ Behavioural & social sciences ☐ Ecological, evolutionary & environmental sciences

For a reference copy of the document with all sections, see nature.com/documents/nr-reporting-summary-flat.pdf

Life sciences study design

All studies must disclose on these points even when the disclosure is negative.

Sample size	The analyses performed in this manuscript are based on a sample size of 210 lepidopteran genomes and 4 trichopteran genomes. This sampling was determined by the availability of chromosome-level reference genomes on INSDC on 27th June 2022.
Data exclusions	Two chromosome-level reference genomes were excluded from our analysis due to quality issues. The first, Zerene cesonia (GCA 012273895.2), contained 246 unlocalised scaffolds that contained 351 BUSCOs. The high number of BUSCOs in these scaffolds means that erroneous rearrangement events would be inferred if this genome were to be included. In the second, Cnaphalocrocis medinalis (GCA 014851415.1), the majority of genes belonging to the M30 Merian element were present on unlocalised scaffolds. We identified two additional genomes that contained minor misassembly issues that we were able to address prior to downstream analysis. In Dendrolimus kikuchii (GCA 019925095.1), we found three scaffolds with a high proportion of duplicated BUSCOs (the majority of which corresponded to the M30 Merian element), indicating that they represented haplotypic duplication. When we removed these scaffolds from the assembly, we successfully recovered a fusion between M30 and MZ that would have otherwise been missed. In Spodoptera frugiperda (GCA 011064685.2), we removed an unlocalised scaffold that contained 22 BUSCOs prior to downstream analyses to avoid inferring a fission event in this species due to assembly issues.
Replication	Our findings were replicated by performing two independent analyses that arrived at the same results for the inference of chromosome

Replication	rearrangements. Each analysis was performed once as replication was not relevant. Otherwise, as this was a comparative genomic study which drew upon all publicly-available chromosome-level reference genomes for Lepidoptera and Trichoptera (with the exception of the two genomes described above) and so using replicates was not relevant.
Randomization	Randomisation was not relevant to this study as we analysed all publicly-available chromosome-level reference genomes for Lepidoptera and Trichoptera (with the exception of the two genomes described above).
Blinding	As above, blinding was not relevant to this study as we analysed all publicly-available chromosome-level reference genomes for Lepidoptera and Trichoptera (with the exception of the two genomes described above). Blinding is also not relevant as we did not carry out a randomised control trial.

Reporting for specific materials, systems and methods

We require information from authors about some types of materials, experimental systems and methods used in many studies. Here, indicate whether each material, system or method listed is relevant to your study. If you are not sure if a list item applies to your research, read the appropriate section before selecting a response.

Materials & experimental systems

n/a	Involved in the study	
☒	☐	Antibodies
☒	☐	Eukaryotic cell lines
☒	☐	Palaeontology and archaeology
☒	☐	Animals and other organisms
☒	☐	Clinical data
☒	☐	Dual use research of concern
☒	☐	Plants

Methods

n/a	Involved in the study	
☒	☐	ChIP-seq
☒	☐	Flow cytometry
☒	☐	MRI-based neuroimaging
